# Supplementary material for: Validity, reliability, and readability of single-item and short physical activity questionnaires for use in surveillance: A systematic review
Source: PLoS One. 2024 Mar 12;19(3):e0300003. doi: 10.1371/journal.pone.0300003 (PMC10931432; doi:10.1371/journal.pone.0300003)
Supplement: S1 File — (DOCX) [file pone.0300003.s001.docx]

S1 File. Blank for studies quality assessment.

| **Question** | | Yes | No | Don’t know/ Comment |
| --- | --- | --- | --- | --- |
| Introduction | | | | |
| 1 | Were the aims/objectives of the study clear? |  |  |  |
| Methods | | | | |
| 2 | Was the study design appropriate for the stated aim(s)? |  |  |  |
| 3 | Was the sample size justified? (through calculation or guidelines) |  |  |  |
| 4 | Was the target/reference population clearly defined? (Is it clear who the research was about?) |  |  |  |
| 5 | Was the sample frame taken from an appropriate population base so that it closely represented the target/reference population under investigation? (Description of selection criteria) |  |  |  |
| 6 | Was the selection process likely to select subjects/participants that were representative of the target/reference population under investigation? |  |  |  |
| 7 | Were observers blind to participants characteristics/previous findings/other observes findings? |  |  |  |
| 8 | Was the examiners experience described? |  |  |  |
| 9 | Was the process of the measurement properties investigation (gold or reference standard for validity; timeline; etc.) described? |  |  |  |
| 1 | Is it clear what was used to determined statistical significance?   1. for reliability: intraclass correlation coefficient or k (inter-measurement reliability) with confidence intervals 2. for validity: correlation coefficient or agreement stats with confidence intervals) |  |  |  |
| 11 | Were the methods (including statistical methods) sufficiently described to enable them to be repeated? |  |  |  |
| Results | | | | |
| 12 | Were the basic data adequately described?  (appropriate descriptive statistics presented (frequencies)) |  |  |  |
| 13 | Was time interval/test-retest procedure described?  (participants' characteristics stable during study period?) |  |  |  |
| 14 | Was information about non-responders described? |  |  |  |
| 15 | Were the results internally consistent? |  |  |  |
| 16 | Were the results presented for all the analyses described in the methods? |  |  |  |
| Discussion | | | | |
| 17 | Were the authors' discussions and conclusions justified by the results? |  |  |  |
| 18 | Were the limitations of the study discussed? |  |  |  |
|  | Summary score |  | | |
