# Supplementary material for: Validity, reliability, and readability of single-item and short physical activity questionnaires for use in surveillance: A systematic review
Source: PLoS One. 2024 Mar 12;19(3):e0300003. doi: 10.1371/journal.pone.0300003 (PMC10931432; doi:10.1371/journal.pone.0300003)
Supplement: S1 Table — (DOCX) [file pone.0300003.s002.docx]

S1 Table. Studies overview.

| **First author, year** | **Country** | **Questionnaire** | **Population assessed** | **Person conducting assessment (self or interview)** | **Measurement properties** | **Measures to compare validity** | **Reliability** **test/retest interval** | **Quality assessment score** |
| --- | --- | --- | --- | --- | --- | --- | --- | --- |
| Ball, 2015 [1] | USA | PAVS and SNAP | 45 adults (clinic staff) | Interviewer | Validity | Accelerometer | NM | 13 |
| Bauman, 2022 [2] | New Zealand | SI-days (Bauman, 2022 Question) | 13887 adults | Interviewer | Validity | IPAQ, single question hours of PA per week | NM | 11 |
| Blomqvist, 2020 [3] | Sweden | SR-1 and SR-2 | 106 adults with chronic heart failure | Responder | Validity, Sensitivity, Specificity | Accelerometer | NM | 12 |
| Cruz, 2021 [4] | Portugal | BPAAT | 110 adults with COPD | Responder | Validity, Sensitivity, Specificity | Accelerometer | NM | 13 |
| Danquah, 2018 [5] | Denmark | NPAQ-short open-ended and NPAQ-short closed-ended | 122 adults | Responder | Validity, Reliability, Sensitivity, Specificity | Accelerometer | 2 weeks | 15 |
| Gill 2012 [6] | USA | Absolute PA Question and Relative PA Question | 159 older adults (mean age 80 years +- 3.9) | Interviewer | Validity, Reliability | Clinical assessment of balance, gait, fear of falling, etc. | 7 days | 14 |
| Gionet, 1989 [7] | Canada | GCSSI and Elaborative Exercise Questionnaire | 551 adults | Interviewer | Validity | VO2max, BMI, ME | NM | 11 |
| Graff-Iversen, 2008 [8] | Norway | Gothenburg instrument, CONOR Instrument | 1497 adults | Interviewer | Validity | IPAQ-L, VO2max, BMI, Triglycerides, HDL-Cholesterol, Waist-to-hip ratio | 3 Years | 10 |
| Hamilton, 2012 [9] | Australia | SIPAM - Parent Version | 458 adults pedometer n=30 | Interviewer | Validity | Pedometer, 7-day PAR | NM | 12 |
| Hart, 2022[10] | USA | PA questions of Brief Health, Fitness and Spirituality Survey | 56 adults | Self-administered | Validity | BMI, fitness question | NM | 10 |
| Hyvärinen, 2020 [11] | Finland | SR-PA L7 | 795 adult women | Self-administered | Validity, Reliability | Accelerometer | 120 days | 14 |
| Iwai, 2001 [12] | Japan | JACC Questionnaire | 1,730 adults for validity and 1,075 adults for reliability | Interviewer | Validity, Reliability | Interview similar to the Minnesota LTPA questionnaire | 1 Year | 13 |
| Jackson, 2007 [13] | USA | PA5, PA8 | 687 Adults | NR | Validity | Clinical assessment, maximal TT and MHPW | NM | 10 |
| Johansson, 2008 [14] | Netherlands | WLPAQ | 9 adults with rheumatoid arthritis | Self-administered | Validity | DLW | NM | 7 |
| Kiernan, 2013 [15] | USA | L-CAT | 267 overweight/obese women | Self-administered | Validity, Reliability | Pedometer and BMI | 2-6 Weeks | 11 |
| Li, 2000 [16] | USA | Usual PA Scale | 188 adult women | Self-reported | Validity | Sturgeon 1993 PAQ, BMI, psychosomatic symptoms, perimenopause-related quality of life. | 14 days | 12 |
| Macdonald, 2022 [17] | Canada | SIPAM | 205 older adults (60 year and older) | Self-reported | Validity | CHAMPS questionnaire | NM | 13 |
| Marshall, 2004 [18] | Australia | BPAAT | 75 adults | Interview | Validity, Reliability | CSA Accelerometer | 7 days | 13 |
| Milton, 2011 [19] | UK | SIPAM, SIPAM - month | 480 adults | Self-reported | Validity, Reliability | SIPAM - GPAQ, SIPAM - month - APS | 7 days | 14 |
| Milton, 2013 [20] | UK | SIPAM | 66 adults | Self-reported | Validity, Specificity, Sensitivity | Accelerometer | NM | 12 |
| Moreno-Llamas, 2020 [21] | Spain | Eurobarometer Survey Question | 39,379 adults | Self-reported | Validity | IPAQ | NM | 7 |
| O'Halloran, 2020 [22] | Australia | SIPAM | 120 adults | NR | Reliability  Validity, specificity, sensitivity | Accelerometer | NM | 14 |
| Orrell, 2007 [23] | UK | TAM1 TAM2 | 73 adults with CHD | Questionnaires | Validity, Reliability | Accelerometer | 7 days | 15 |
| Portegijs, 2017 [24] | Finland | Self-report scale to assess habitual physical activity | 848 adults aged 75-90 years  (174 for accelerometer data) | Interviewer | Validity | Accelerometer 15-item life space assessment Walking difficulty question  Short Physical Performance Battery |  | 12 |
| Puig Ribera, 2012 [25] | Spain | Catalonian and Spanish versions of BPAAT | 105 adults | Interview | Validity, Reliability | IPAQ-SF | 14-28 Days | 15 |
| Puig-Ribera, 2015 [26] | Spain | Spanish BPAAT | 1184 adults | Interviewer | Validity | 7-day Physical Activity Recall (7DPAR) Accelerometer | NM | 16 |
| Rose, 2008 [27] | New Zealand | Single-item screening question | 1171 women | Interviewer | Validity, Sensitivity, Specificity | New Zealand Physical Activity Questionnaire - Long Form (NZPAQ-LF) | NM | 11 |
| Ross, 2018 [28] | USA | L-CAT | 76 adults with obesity | Responder (Online) | Validity, Sensitivity, Specificity | Accelerometer | NM | 15 |
| Schechtman, 1991 [29] | USA | St. Louis Working Hearts Program single exercise question | 1004 adults | NR | Validity | BMI, HDL Cholesterol, oxygen capacity | NM | 7 |
| Smith, 2005 [30] | Australia | BPAAT 3QA | 509 adults | Interviewer | Validity, Reliability | Active Australia Questionnaire, Accelerometer | 3 days | 17 |
| Taylor-Piliae, 2006 [31] | USA | SBAS | 1,010 adults | Self-administered | Validity | Stanford Seven Day Physical Activity Recall, cardiovascular disease risk biomarkers | NM | 14 |
| Webster, 2011 [32] | Australia | The six-point scale | 100 adults | Self-administered | Validity | IPAQ HAP Questionnaire | NM | 14 |
| Weiss, 1990 [33] | USA | Job Related Activity  Main Daily Activity  Compared to Peers Question | 33,630 adults | Interview | Validity | Continuous energy expenditure measure | NM | 10 |
| Yore, 2006 [34] | USA | BRFSS | 5,847 adults | Interview | Validity | OPAQ | NM | 12 |
| Zwolinsky, 2015 [35] | England | SIPAM | 7650 adults | Self-reported | % Agreement, Sensitivity, Specificity | IPAQ | NM | 14 |

NM=not measured, NR=not reported, PA=physical activity, PAVS=Physical Activity and Vital Signs, SNAP=Speedy Nutrition and Physical Activity Assessment, SI-Days=single-item day question, IPAQ= International Physical Activity Questionnaire, SR-1=Self-report on activity 1, SR-2=Self-report on activity 2, BPAAT= Brief Physical Activity Assessment Tool, COPD= chronic obstructive pulmonary disorder, NPAQ=Nordic physical activity questionnaire, PA=physical activity, GCSSI=Godin and coworkers simple self-administered instrument, BMI=body mass index, ME=muscular endurance, CONOR=Cohort of Norway, IPAQ-L=International Physical Activity Questionnaire – Long Form, HDL=high density lipoproteins, SIPAM=Single Item Physical Activity Measure, PAR=physical activity recall, SR-PA L7=The Seven-level Single-Question Scale for Self-Reported Leisure Time Physical Activity, JACC=Japan Collaborative Cohort, LTPA=leisure time physical activity, PA5=single-response from five descriptors, PA8=single-response from eight descriptors, WLPAQ=Work Leisure Physical Activity Questionnaire, DLW=Doubly-labeled water, L-CAT=The Stanford Leisure-Time Activity Categorical Item, PAQ=physical activity questionnaire, CHAMPS=Community Health Activities Model Program for Seniors physical activity self-report questionnaire, CSA=Computer Science Application, GPAQ=Global Physical Activity Questionnaire, APS=Active People Survey, TAM=Total Activity Measure, CHD=Coronary Heart Disease, 7DPAR=7-day physical activity recall, NZPAQ-LF=New Zealand physical activity questionnaire-Long form, SBAS=Stanford Brief Activity Survey, HAP=Human activity profile, BRFSS=Behavioral Risk Factor Surveillance System, OPAQ=Occupational Physical Activity Questionnaire

1. Ball TJ, Joy EA, Goh TL, Hannon JC, Gren LH, Shaw JM. Validity of two brief primary care physical activity questionnaires with accelerometry in clinic staff. Primary Health Care Research & Development. 2015;16(1):100-8. doi: DOI: 10.1017/S1463423613000479.

2. Bauman AE, Richards JA. Understanding of the Single-Item Physical Activity Question for Population Surveillance. Journal of Physical Activity and Health. 2022;19(10):681-6. doi: 10.1123/jpah.2022-0369.

3. Blomqvist A, Bäck M, Klompstra L, Strömberg A, Jaarsma T. Utility of single-item questions to assess physical inactivity in patients with chronic heart failure. ESC Heart Fail. 2020;7(4):1467-76. doi: 10.1002/ehf2.12709.

4. Cruz J, Jácome C, Oliveira A, Paixão C, Rebelo P, Flora S, et al. Construct validity of the brief physical activity assessment tool for clinical use in COPD. Clin Respir J. 2021;15(5):530-9. doi: 10.1111/crj.13333.

5. Danquah IH, Petersen CB, Skov SS, Tolstrup JS. Validation of the NPAQ-short - A brief questionnaire to monitor physical activity and compliance with the WHO recommendations. BMC Public Health. 2018;18(1). doi: 10.1186/s12889-018-5538-y.

6. Gill DP, Jones GR, Zou G, Speechley M. Using a single question to assess physical activity in older adults: A reliability and validity study. BMC Medical Research Methodology. 2012;12(20). doi: 10.1186/1471-2288-12-2022373159.

7. Gionet NJ, Godin G. Self-Reported Exercise Behavior of Employees - A Validity Study. Journal of Occupational and Environmental Medicine. 1989;31(12):969-73. doi: 10.1097/00043764-198912000-00007.

8. Graff-Iversen S, Anderssen SA, Holme IM, Jenum AK, Raastad T. Two short questionnaires on leisure-time physical activity compared with serum lipids, anthropometric measurements and aerobic power in a suburban population from Oslo, Norway. European journal of epidemiology. 2008;23(3):167-74. doi: 10.1007/s10654-007-9214-2.

9. Hamilton K, White KM, Cuddihy T. Using a single-item physical activity measure to describe and validate parents’ physical activity patterns. Research Quarterly for Exercise and Sport. 2012;83(2):340-5. doi: 10.1080/02701367.2012.10599865.

10. Hart PD. Initial Assessment of a Brief Health, Fitness, and Spirituality Survey for Epidemiological Research: A Pilot Study. J Lifestyle Med. 2022;12(3):119-26. doi: 10.15280/jlm.2022.12.3.119. PubMed PMID: 36628178; PubMed Central PMCID: PMCPMC9798877.

11. Hyvärinen M, Sipilä S, Kulmala J, Hakonen H, Tammelin TH, Kujala UM, et al. Validity and reliability of a single question for leisure-time physical activity assessment in middle-aged women. Journal of Aging and Physical Activity. 2020;28(2):231-41. doi: 10.1123/JAPA.2019-0093.

12. Iwai N, Hisamichi S, Hayakawa N, Inaba Y, Nagaoka T, Sugimori H, et al. Validity and reliability of single-item questions about physical activity. Journal of Epidemiology. 2001;11(5):211-8. doi: 10.2188/jea.11.211.

13. Jackson AW, Morrow JR, Jr., Bowles HR, Fitzgerald SJ, Blair SN. Construct validity evidence for single-response items to estimate physical activity levels in large sample studies. Research Quarterly for Exercise and Sport. 2007;78(2):24-31. doi: 10.1080/02701367.2007.10599400.

14. Johansson G, Westerterp KR. Assessment of the physical activity level with two questions: Validation with doubly labeled water. International Journal of Obesity. 2008;32(6):1031-3. doi: 10.1038/ijo.2008.42.

15. Kiernan M, Schoffman DE, Lee K, Brown SD, Fair JM, Perri MG, et al. The stanford leisure-time activity categorical item (L-Cat): A single categorical item sensitive to physical activity changes in overweight/obese women. International Journal of Obesity. 2013;37(12):1597-602. doi: 10.1038/ijo.2013.36.

16. Li S, Carlson E, Holm K. Validation of a single-item measure of usual physical activity. Perceptual and Motor Skills. 2000;91(2):593-602. doi: 10.2466/pms.2000.91.2.593.

17. Macdonald HM, Nettlefold L, Bauman A, Sims-Gould J, McKay HA. Pragmatic Evaluation of Older Adults' Physical Activity in Scale-Up Studies: Is the Single-Item Measure a Reasonable Option? Journal of Aging and Physical Activity. 2022;30(1):25-32. doi: 10.1123/japa.2020-0412.

18. Marshall AL, Smith BJ, Bauman AE, Kaur S. Reliability and validity of a brief physical activity assessment for use by family doctors. British Journal of Sports Medicine. 2005;39(5):294-7. doi: 10.1136/bjsm.2004.013771.

19. Milton K, Bull FC, Bauman A. Reliability and validity testing of a single-item physical activity measure. British Journal of Sports Medicine. 2011;45(3):203-8. doi: 10.1136/bjsm.2009.068395.

20. Milton K, Clemes S, Bull F. Can a single question provide an accurate measure of physical activity? British Journal of Sports Medicine. 2013;47(1):44-8. doi: 10.1136/bjsports-2011-090899.

21. Moreno-Llamas A, García-Mayor J, De la Cruz-Sánchez E. Concurrent and convergent validity of a single, brief question for physical activity assessment. International Journal of Environmental Research and Public Health. 2020;17(6):1989. doi: 10.3390/ijerph17061989.

22. O’Halloran P, Kingsley M, Nicholson M, Staley K, Randle E, Wright A, et al. Responsiveness of the single item measure to detect change in physical activity. PLoS ONE. 2020;15(6). doi: 10.1371/journal.pone.0234420.

23. Orrell A, Doherty P, Miles J, Lewin R. Development and validation of a very brief questionnaire measure of physical activity in adults with coronary heart disease. European Journal of Cardiovascular Prevention and Rehabilitation. 2007;14(5):615-23. doi: 10.1097/HJR.0b013e3280ecfd56.

24. Portegijs E, Sipilä S, Viljanen A, Rantakokko M, Rantanen T. Validity of a single question to assess habitual physical activity of community-dwelling older people. Scandinavian Journal of Medicine & Science in Sports. 2017;27(11):1423-30. doi: 10.1111/sms.12782.

25. Puig Ribera A, Peña Chimenis Ò, Romaguera Bosch M, Duran Bellido E, Heras Tebar A, Solà Gonfaus M, et al. How to identify physical inactivity in Primary Care: Validation of the Catalan and Spanish versions of 2 short questionnaires. Atencion Primaria. 2012;44(8):485-93. doi: 10.1016/j.aprim.2012.01.005.

26. Puig-Ribera A, Martín-Cantera C, Puigdomenech E, Real J, Romaguera M, Magdalena-Belio JF, et al. Screening physical activity in family practice: Validity of the Spanish version of a brief physical activity questionnaire. PLoS ONE. 2015;10(9). doi: 10.1371/journal.pone.0136870.

27. Rose SB, Elley CR, Lawton BA, Dowell AC. A single question reliably identifies physically inactive women in primary care. The New Zealand Medical Journal. 2008;121(1268):U2897.

28. Ross KM, Leahey TM, Kiernan M. Validation of the Stanford Leisure-Time Activity Categorical Item (L-Cat) using armband activity monitor data. *Obesity science & practice*. 2018;4(3):276-82. doi: 10.1002/osp4.155.

29. Schechtman KB, Barzilai B, Rost K, B. FJE. Measuring physical activity with a single question. American journal of public health. 1991;81(6):771-3.

30. Smith BJ, Marshall AL, Huang N. Screening for physical activity in family practice: Evaluation of two brief assessment tools. American journal of preventive medicine. 2005;29(4):256-64. doi: 10.1016/j.amepre.2005.07.005.

31. Taylor-Piliae RE, Norton LC, Haskell WL, Mahbouda MH, Fair JM, Iribarren C, et al. Validation of a new brief physical activity survey among men and women aged 60-69 years. American Journal of Epidemiology. 2006;164(6):598-606. doi: 10.1093/aje/kwj248.

32. Webster S, Khan A, Nitz JC. A brief questionnaire is able to measure population physical activity levels accurately: A comparative validation study. Journal of Clinical Gerontology and Geriatrics. 2011;2(3):83-7. doi: 10.1016/j.jcgg.2011.06.003.

33. Weiss TW, Slater CH, Green LW, Kennedy VC, Albright DL, Wun CC. The validity of single-item, self-assessment questions as measures of adult physical activity. Journal of Clinical Epidemiology. 1990;43(11):1123-9. doi: 10.1016/0895-4356(90)90013-F.

34. Yore MM, Bowles HR, Ainsworth BE, Macera CA, Kohl HW, III. Single versus multiple item questions on occupational physical activity. Journal of Physical Activity and Health. 2006;3(1):102-11. doi: 10.1123/jpah.3.1.102.

35. Zwolinsky S, McKenna J, Pringle A, Widdop P, Griffiths C. Physical activity assessment for public health: Efficacious use of the single-item measure. Public Health. 2015;129(12):1630-6. doi: 10.1016/j.puhe.2015.07.015.
