# Supplementary material for: Validity, reliability, and readability of single-item and short physical activity questionnaires for use in surveillance: A systematic review
Source: PLoS One. 2024 Mar 12;19(3):e0300003. doi: 10.1371/journal.pone.0300003 (PMC10931432; doi:10.1371/journal.pone.0300003)
Supplement: S2 Table — (DOCX) [file pone.0300003.s003.docx]

S2 Table. Summary of measurement properties.

| **Questionnaire** | **Reliability** | **Validity** | **Sensitivity** | **Specificity** | **Identification of the population meeting WHO recommendations for the minimum duration of PA** |
| --- | --- | --- | --- | --- | --- |
| Number days of sufficient amount of PA per week | | | | | |
| Physical Activity and Vital Signs  (PAVS) (1) | NM | Total weekly min MVPA  Bout PA rP=0.50 Non-Bout rP=0.33 No. of days >30min MVPA Bout rP=0.52 Non-bout rP=0.30 | NM | NM | kappa=0.46 (95%CI: 0.04-0.89) |
| Single Item Physical Activity Measure  (SIPAM) | kappa=0.63 (95%CI: 0.54-0.72)  Rho=0.72  (2) | Rho=0.53  (2) |  |  |  |
|  |  | Total MVPA Rho=0.46 MVBA Bouts Rho=0.57  (3) | Total MVPA: 48% BoutsMVPA: 83%  (3) | Total MVPA: 88% BoutsMVPA: 56%  (3) | Identifying meeting the guidelines kappa=0.13 (95%CI: 0.12-0.14) Classification of inactive participants  kappa=0.45 (95%CI: 0.43-0.47)  (3) |
|  |  |  | Identifying meeting the guidelines: 18.7% Classification of inactive participants: 74.2% (4) | Identifying meeting the guidelines: 97.2% Classification of inactive participants: 79.7%  (4) | Identifying meeting the guidelines: kappa=0.13 (95%CI: 0.12-0.14) Classification of inactive participants  kappa=0.45 (95%CI: 0.43-0.47)  (4) |
|  |  | MVPAtotal Rho=0.22 MVPAbouts Rho=0.51 MVPAtotalchange Rho=0.36 MVPAboutschange Rho=0.40  (5) | MVPAtotal= 63.1% (95%CI: 50.2-74.7%) MVPAbouts=69.2% (95%CI: 56.6-80.1%)  (5) | MVPAtotal=52.0% (95%CI: 31.3-72.2%) MVPAbouts=48.0% (95%CI: 21.2-68.7%)  (5) |  |
|  |  | Rho=0.33-0.55 (6) |  |  |  |
|  |  | AUC-ROC: against question hours per week 0.82 (95%CI: 0.81-0.83); against IPAQ total 0.71 (95%CI: 0.7-0.72); against IPAQ LTPA 0.75 (95%CI: 0.74-0.76)  (7) |  |  |  |
|  |  | Version for parents:  7-day PAR rP=0.51 Pedometer rP=0.81  (8) |  |  |  |
|  | Month version:  kappaw=0.76 (95%CI 0.69-0.82)  Rho=0.82  (2) | Month version:  Rho=0.33-0.48  (2) |  |  |  |
| Brief Physical Activity Assessment Tool  (BPAAT) | Rho=0.61 (95%CI: 0.53-0.69) kappa=0.58 (95%CI: 0.47-0.69)  (9) | AAQ Reference Rho=0.54 (95%CI: 0.44-0.63) kappa=46.7% (95%CI: 35.6%-57.9%) Accelerometer Rho=0.39 (95%CI: 0.28-0.49) kappa=0.18 (95%CI: 0.39-0.33) (9) |  |  |  |
|  | kappa=0.53 (95%CI: 0.33-0.72)  (10) | kappa =0.40 (95%CI: 0.12-0.69)  (10) |  |  |  |
|  |  | rho=0.394-0.435  (11) | 50-52%  (11) | 84-91%  (11) |  |
|  |  | Spanish version:  Against accelerometer:  MPA rP=0.22 (95%CI: 0.156-0.272) VPA rP=0.28  (95%CI:0.165-0.391) Agreement with 7DPAR Kappa=0.454 (95%CI: 0.405-0.505) AUC-ROC: 0.8 (95%CI: 0.78-0.83)  (12) | Spanish version:  0.75 (95%CI: 0.713-0.773)  (12) | Spanish version:  0.74 (95%CI: 0.702-0.790)  (12) |  |
|  | Spanish version:  kappa=0.70 (95%CI: 0.53-0.82)  (13) | Spanish version:  kappa=0.64 (95%CI: 0.50-0.81)  (13) |  |  |  |
|  | Catalan version:  kappa=0.72 (95%CI: 0.55-0.83)  (13) | Catalan version:  kappa=0.58 (95%CI: 0.43-0.77)  (13) |  |  |  |
| Three-Question Assessment  (3QA)  (9) | Rho=0.63 (95%CI: 0.53-0.70) kappa=0.56 (95%CI: 0.44-0.67) | AAQ Reference Rho=0.43 (95%CI: 0.32-0.53) kappa=38.7% (95%CI: 26.4%-51.1%) Accelerometer  Rho=0.31 (95%CI: 0.18-0.43) kappa=24.3% (95%CI: 11.6%-36.9%) |  |  |  |
| Amount of PA | | | | | |
| Elaborative Exercise Questionnaire (14) |  | rP=0.18 and lower |  |  |  |
| Self-report on Activity 2  (SR-2) (15) |  | rho=0.21 | 60% (Youden method) | 54% (Youden method) |  |
| JACC Questionnaire (16) | kappaw=0.39 - 0.56 | Rho=0.43-0.60 |  |  |  |
| Nordic Physical Activity Questionnaire  (NPAQ-short)  (17) | Open ended:  MVPA rho=0.82 VPA rho=0.80 | MVPA rho=0.33 VPA rho=0.32 | 90% | 50% | kappa=0.42 |
|  | Closed-ended:  MVPA kappa=0.66 VPA kappa=0.59 | MVPA kappa=0.17 VPA kappa=0.21 | 81% | 55% | kappa=0.34 |
| Total Activity Measure  (TAM) (18) | TAM 1:  Rho=0.73 (95%CI: 0.56-0.83) | rP=0.257 |  |  |  |
|  | TAM 2:  Rho=0.82 (95%CI: 0.71-0.88) | rP=0.36-0.38 |  |  |  |
| Cohort of Norway  (CONOR) Instrument (19) |  | For objective measures (VO2max, BMI, Triglycerides, HDL-Cholesterol, Waist to hip ratio) Rho=-0.14-0.13. Statistically significant increase of MET values IPAQ-L in more active groups. |  |  |  |
| General exercise participation/physical activity involvement questions | | | | | |
| Self-report on Activity 1  (SR-1)  (15) |  | rho=0.24 | 30% | 92% |  |
| Single-item screening question to identify physical inactivity  (20) |  | kappa=0.46-0.56 | 76.7% (95%CI: 73.5-79.7) | 81.1% (95%CI: 77.2-84.4) |  |
| St. Louis Working Hearts Program single exercise question (21) |  | Exercisers had statistically significant lower BMI, greater oxygen capacity in both genders, and woman had a higher HDL cholesterol. |  |  |  |
| PA questions of Brief Health, Fitness and Spirituality Survey (22) |  | Significant correlation with fitness rP=0.426  No significant correlation with BMI |  |  |  |
| Self-reported level of PA compared with peers | | | | | |
| Compared to Peers Question (23) |  | Rho=0.28  Rho=0.29 (with elaboration question) |  |  |  |
| Relative PA Question  (24) | kappaw=0.56 (95%CI: 0.30-0.82) | Rho from 0.28 (95%CI: 0.15-0.44) to 0.57 (95%CI: 0.38-0.78) |  |  |  |
| Categorical descriptions of PA levels | | | | | |
| The Seven-level Single-Question Scale for Self-Reported Leisure Time Physical Activity  (SR-PA L7) (25) | Rho=0.707  Three categories  Rho=0.622 | light PA Rho=0.105,  MVPA Rho=0.318,  total counts Rho=0.333  Three categories  light PA Rho=0.109  MVPA Rho=0.337  total counts Rho=0.333 |  |  |  |
| Self-report scale to assess habitual physical activity  (26) |  | step count Rho=0.49 moderate PA Rho=0.49 Low-Intensity PA Rho=0.40 |  |  |  |
| PA categories (27) |  | PA5:  Partial correlations with controlling for age and gender with TT r=0.57; with MHPW, r=0.44. Participants who according to questionnaire met the recommended PA levels had a lower percentage of body fat and cholesterol.  PA8:  Partial correlations with controlling for age and gender with TT r=0.53; with MHPW, r=0.43. Participants who according to questionnaire met the recommended PA levels had a lower percentage of body fat and cholesterol. |  |  |  |
| Absolute PA Question  (24) | kappaw=0.75 (95%CI: 0.60-0.91) | Rho from 0.10 (95%CI: 0.00-0.26) to 0.33 (95%CI: 0.19-0.49) |  |  |  |
| Job Related Activity  (23) |  | Rho=-0.02 |  |  |  |
| Main Daily Activity  (23) |  | Rho=0.22 |  |  |  |
| Godin and coworkers simple self-administered instrument  (GCSSI)  (14) |  | rP=0.4 and lower |  |  |  |
| Usual PA Scale  (28) | Rho=0.88 Internal consistency reliability (Correction for attenuation method Rho=0.68) | Against the PA questionnaire Rho=0.66. |  |  |  |
| The Stanford Leisure-Time Activity Categorical Item  (L-CAT) |  | Against Steps Rho=0.38-0.41, weekly minutes MVPA Rho=0.38-0.40, Weekly minutes of MVPA bouts Rho=0.38-0.4  (29) | baseline: 30.4% 6mo: 54.2%  (29) | baseline: 88.7% 6mo: 74.5%  (29) |  |
|  | Rho=0.80, Kappaw=0.64 (95%CI: 0.54-0.73)  (30) | Linear regression for BMI: An increase in L-Cat category was associated with a lower BMI at baseline of ~0.5 BMI unit. (B= -0.4 (95%CI: -0.8 to -0.1; B= -0.14; P=0.02). Linear regression for Pedometer steps at 6 months: An increase in one L-Cat category was associated with 1059 more steps (95%CI: 712-1407; B= 0.38; P<0.001).  (30) |  |  |  |
| Work Leisure Physical Activity Questionnaire  (WLPAQ) (31) |  | Bland-Altman plot showed small difference between EE measured by questionnaire and with DLW. Highest r value with the mean values=0.89. |  |  |  |
| Speedy Nutrition and Physical Activity Assessment (SNAP)  (1) |  | Total weekly min MVPA  Bout PA Rho=0.32 Non-Bout Rho=0.41 No. of days >30min MVPA Bout Rho=0.31 Non-bout Rho=0.49 |  |  | kappa=0.12(95%CI: 0.04-0.28) |
| Stanford Brief Activity Survey (SBAS)  (32) |  | Higher active categories in SBAS have statistically significant higher minutes per week and EE per day (Stanford Seven-day Physical activity recall) and lower cardiovascular disease risk biomarkers. |  |  |  |
| The six-point scale  (33) |  | IPAQ kappa=0.46 (95%CI: 0.26-0.66) HAP kappa=0.57 (95%CI: 0.41-0.73) IPAQ Rho=0.49 HAP Rho=0.69 |  |  |  |
| Occupational Physical Activity Question from the Behavioral Risk Factor Surveillance System  (BRFSS)  (34) |  | kappa=0.56(95%CI: 0.54-0.58) |  |  |  |
| Eurobarometer Survey Question (35) |  | AUC-ROC: 0.751 (95%CI: 0.743-0.759) to 0.947 (95%CI: 0.945-0.95) (higher for higher PA level) |  |  |  |
| Gothenburg Instrument (19) |  | For objective measures (VO2max, BMI, Triglycerides, HDL-Cholesterol, Waist to hip ratio) Rho=-0.19-0.16. Statistically significant increase of MET values IPAQ-L in more active groups. |  |  |  |

PA=physical activity, NM=not measured, MVPA=moderate to vigorous physical activity, CI=confidence interval, AUC-ROC=Area under the receiver operating characteristic curve, PAR=physical activity recall, BPAAT=brief physical activity assessment tool, AAQ=active Australia questionnaire, PAVS=physical activity and vital signs, SIPAM=single-item physical activity questionnaire, MPA=moderate physical activity, VPA=vigorous physical activity, 7DPAR=7-day physical activity recall, TAM=total activity measure, BMI=body mass index, IPAQ-L=International physical activity questionnaire long form, HDL=high density lipoprotein, MET=metabolic equivalent, CONOR=Cohort of Norway, SR-1=Self-report on activity 1, SR-PA L7=the Seven-level Single-Question Scale for Self-Reported Leisure Time Physical Activity, PA-5= single-response from five descriptors, PA-8= single-response from eight descriptors, MHPW=MET hours of reported PA per Week, TT=treadmill test, GCSSI=Godin and coworkers simple self-administered instrument, L-CAT=the Stanford Leisure-Time Activity Categorical Item, WLPAQ=Work Leisure Physical Activity Questionnaire, DLW=doubly-labeled water, EE=energy expenditure, IPAQ=International Physical activity questionnaire, HAP=human activity profile, BRFSS=Behavioral Risk Factor Surveillance System

1. Ball TJ, Joy EA, Goh TL, Hannon JC, Gren LH, Shaw JM. Validity of two brief primary care physical activity questionnaires with accelerometry in clinic staff. Primary Health Care Research & Development. 2015;16(1):100-8.

2. Milton K, Bull FC, Bauman A. Reliability and validity testing of a single-item physical activity measure. British Journal of Sports Medicine. 2011;45(3):203-8.

3. Milton K, Clemes S, Bull F. Can a single question provide an accurate measure of physical activity? British Journal of Sports Medicine. 2013;47(1):44-8.

4. Zwolinsky S, McKenna J, Pringle A, Widdop P, Griffiths C. Physical activity assessment for public health: Efficacious use of the single-item measure. Public Health. 2015;129(12):1630-6.

5. O’Halloran P, Kingsley M, Nicholson M, Staley K, Randle E, Wright A, et al. Responsiveness of the single item measure to detect change in physical activity. PLoS ONE. 2020;15(6).

6. Macdonald HM, Nettlefold L, Bauman A, Sims-Gould J, McKay HA. Pragmatic Evaluation of Older Adults' Physical Activity in Scale-Up Studies: Is the Single-Item Measure a Reasonable Option? Journal of Aging and Physical Activity. 2022;30(1):25-32.

7. Bauman AE, Richards JA. Understanding of the Single-Item Physical Activity Question for Population Surveillance. Journal of Physical Activity and Health. 2022.

8. Hamilton K, White KM, Cuddihy T. Using a single-item physical activity measure to describe and validate parents’ physical activity patterns. Research Quarterly for Exercise and Sport. 2012;83(2):340-5.

9. Smith BJ, Marshall AL, Huang N. Screening for physical activity in family practice: Evaluation of two brief assessment tools. American journal of preventive medicine. 2005;29(4):256-64.

10. Marshall AL, Smith BJ, Bauman AE, Kaur S. Reliability and validity of a brief physical activity assessment for use by family doctors. British Journal of Sports Medicine. 2005;39(5):294-7.

11. Cruz J, Jácome C, Oliveira A, Paixão C, Rebelo P, Flora S, et al. Construct validity of the brief physical activity assessment tool for clinical use in COPD. The Clinical Respiratory Journal. 2021;15(5):530-9.

12. Puig-Ribera A, Martín-Cantera C, Puigdomenech E, Real J, Romaguera M, Magdalena-Belio JF, et al. Screening physical activity in family practice: Validity of the Spanish version of a brief physical activity questionnaire. PLoS ONE. 2015;10(9).

13. Puig Ribera A, Peña Chimenis Ò, Romaguera Bosch M, Duran Bellido E, Heras Tebar A, Solà Gonfaus M, et al. How to identify physical inactivity in Primary Care: Validation of the Catalan and Spanish versions of 2 short questionnaires. Atencion Primaria. 2012;44(8):485-93.

14. Gionet NJ, Godin G. Self-Reported Exercise Behavior of Employees - A Validity Study. Journal of Occupational and Environmental Medicine. 1989;31(12):969-73.

15. Blomqvist A, Bäck M, Klompstra L, Strömberg A, Jaarsma T. Utility of single-item questions to assess physical inactivity in patients with chronic heart failure. ESC Heart Fail. 2020;7(4):1467-76.

16. Iwai N, Hisamichi S, Hayakawa N, Inaba Y, Nagaoka T, Sugimori H, et al. Validity and reliability of single-item questions about physical activity. Journal of Epidemiology. 2001;11(5):211-8.

17. Danquah IH, Petersen CB, Skov SS, Tolstrup JS. Validation of the NPAQ-short - A brief questionnaire to monitor physical activity and compliance with the WHO recommendations. BMC Public Health. 2018;18(1).

18. Orrell A, Doherty P, Miles J, Lewin R. Development and validation of a very brief questionnaire measure of physical activity in adults with coronary heart disease. European Journal of Cardiovascular Prevention and Rehabilitation. 2007;14(5):615-23.

19. Graff-Iversen S, Anderssen SA, Holme IM, Jenum AK, Raastad T. Two short questionnaires on leisure-time physical activity compared with serum lipids, anthropometric measurements and aerobic power in a suburban population from Oslo, Norway. European journal of epidemiology. 2008;23(3):167-74.

20. Rose SB, Elley CR, Lawton BA, Dowell AC. A single question reliably identifies physically inactive women in primary care. The New Zealand Medical Journal. 2008;121(1268):U2897.

21. Schechtman KB, Barzilai B, Rost K, B. FJE. Measuring physical activity with a single question. American journal of public health. 1991;81(6):771-3.

22. Hart PD. Initial Assessment of a Brief Health, Fitness, and Spirituality Survey for Epidemiological Research: A Pilot Study. J Lifestyle Med. 2022;12(3):119-26.

23. Weiss TW, Slater CH, Green LW, Kennedy VC, Albright DL, Wun CC. The validity of single-item, self-assessment questions as measures of adult physical activity. Journal of Clinical Epidemiology. 1990;43(11):1123-9.

24. Gill DP, Jones GR, Zou G, Speechley M. Using a single question to assess physical activity in older adults: A reliability and validity study. BMC Medical Research Methodology. 2012;12(20).

25. Hyvärinen M, Sipilä S, Kulmala J, Hakonen H, Tammelin TH, Kujala UM, et al. Validity and reliability of a single question for leisure-time physical activity assessment in middle-aged women. Journal of Aging and Physical Activity. 2020;28(2):231-41.

26. Portegijs E, Sipilä S, Viljanen A, Rantakokko M, Rantanen T. Validity of a single question to assess habitual physical activity of community-dwelling older people. Scandinavian Journal of Medicine & Science in Sports. 2017;27(11):1423-30.

27. Jackson AW, Morrow JR, Jr., Bowles HR, Fitzgerald SJ, Blair SN. Construct validity evidence for single-response items to estimate physical activity levels in large sample studies. Research Quarterly for Exercise and Sport. 2007;78(2):24-31.

28. Li S, Carlson E, Holm K. Validation of a single-item measure of usual physical activity. Perceptual and Motor Skills. 2000;91(2):593-602.

29. Ross KM, Leahey TM, Kiernan M. Validation of the Stanford Leisure-Time Activity Categorical Item (L-Cat) using armband activity monitor data. Obesity science & practice. 2018;4(3):276-82.

30. Kiernan M, Schoffman DE, Lee K, Brown SD, Fair JM, Perri MG, et al. The stanford leisure-time activity categorical item (L-Cat): A single categorical item sensitive to physical activity changes in overweight/obese women. International Journal of Obesity. 2013;37(12):1597-602.

31. Johansson G, Westerterp KR. Assessment of the physical activity level with two questions: Validation with doubly labeled water. International Journal of Obesity. 2008;32(6):1031-3.

32. Taylor-Piliae RE, Norton LC, Haskell WL, Mahbouda MH, Fair JM, Iribarren C, et al. Validation of a new brief physical activity survey among men and women aged 60-69 years. American Journal of Epidemiology. 2006;164(6):598-606.

33. Webster S, Khan A, Nitz JC. A brief questionnaire is able to measure population physical activity levels accurately: A comparative validation study. Journal of Clinical Gerontology and Geriatrics. 2011;2(3):83-7.

34. Yore MM, Bowles HR, Ainsworth BE, Macera CA, Kohl HW, III. Single versus multiple item questions on occupational physical activity. Journal of Physical Activity and Health. 2006;3(1):102-11.

35. Moreno-Llamas A, García-Mayor J, De la Cruz-Sánchez E. Concurrent and convergent validity of a single, brief question for physical activity assessment. International Journal of Environmental Research and Public Health. 2020;17(6):1989.
