# Supplementary material for: Validity, reliability, and readability of single-item and short physical activity questionnaires for use in surveillance: A systematic review
Source: PLoS One. 2024 Mar 12;19(3):e0300003. doi: 10.1371/journal.pone.0300003 (PMC10931432; doi:10.1371/journal.pone.0300003)
Supplement: S3 Table — (DOCX) [file pone.0300003.s004.docx]

S3 Table. Length and readability levels of short physical activity questionnaires.

| **Tool** | **Text** | **Word Count** | **Flesch Reading Score*** | **Flesch-Kincaid Grade Level*** | **Self-administered Reading Time (seconds)** | **Interviewer Speaking Time (seconds)** |
| --- | --- | --- | --- | --- | --- | --- |
| Number days of sufficient amount of PA per week | | | | | | |
| Physical Activity and Vital Signs (PAVS) | 1 How many days in the past week have you performed PA where your heart beats faster and your breathing is harder than normal for 30 minutes or more? (in 3 times 10 minute bouts, or 1 time 30 minutes bout) *0 1 2 3 4 5 6 7 (circle one)* 2 How many days in a typical week have you performed activity such as this?  *0 1 2 3 4 5 6 7 (circle one)* | 76 | 80,22 | 6th grade (easy to read) | 19 | 24 |
| Single-Item Physical Activity Measure (SIPAM) | In the past week, on how many days have you done a total of 30 minutes or more of physical activity, which was enough to raise your breathing rate? This may include sport, exercise, and brisk walking or cycling for recreation or to get to and from places, but should not include housework or physical activity that may be part of your job. | 63 | 55,35 | 10th to 12th grade (fairly difficult to read) | 16 | 21 |
|  | Month variation: In the past month, on how many days have you done a total of 30 minutes or more of physical activity, which was enough to raise your breathing rate? This may include sport, exercise, and brisk walking or cycling for recreation or to get to and from places, but should not include housework or physical activity that may be part of your job. | 63 | 55,35 | 10th to 12th grade (fairly difficult to read) | 16 | 21 |
|  | Bauman variation: In the past week, on how many days have you done a total of 30 minutes or more of physical activity, which was enough to raise your breathing rate? This may include sport, traditional games, kapa haka, exercise and brisk walking or cycling for recreation or to get to and from places, but should not include housework or physical activity that may be part of your job. | 67 | 49,09 | College (difficult to read) | 17 | 22 |
|  | *Parents version*: On how many days in the course of the past week (past 7 days) have you engaged in at least 15-30 minutes of at least a moderate-intensity PA? (any activity that makes you breathe somewhat harder than normal, e.g., brisk walking, playing energetic games with children, sweeping, cleaning gutters, regular paced swimming or cycling, low-impact aerobics) to activities that make you breathe much harder than normal (e.g., heavy manual labour, moving heavy furniture, playing strenuous games with the children, high impact aerobics, running, fast bicycling) of which could be built up during the day with a variety of activities of at least 10 minutes in length, or done in one session. | 111 | 18,65 | College graduate (very difficult to read) | 28 | 36 |
| Brief Physical Activity Assessment Tool  (BPAAT) | 1 How many times a week do you usually do 20 minutes or more of vigorous-intensity physical activity that makes you sweat or puff and pant? (e.g., heavy lifting, digging, jogging, aerobics, or fast bicycling). *3 or more times a week.* *1 to 2 times a week.* *None.* 2 How many times a week do you usually do 30 minutes or more of moderate-intensity physical activity or walking that increases your heart rate or makes you breathe harder than normal? (e.g., carrying light loads, bicycling at a regular pace, or playing doubles tennis). *5 or more times a week.* *3–4 times a week.* *1–2 times a week.* *None.* | 106 | 49,06 | College (difficult to read) | 27 | 35 |
| Three-Question Assessment  (3QA) | 1 How many times a week do you usually do 20 minutes or more of vigorous-intensity physical activity that makes you sweat or puff and pant? (e.g., heavy lifting, digging, jogging, aerobics, or fast bicycling)? *3 or more times a week* *1 to 2 times a week* *none* 2 How many times a week do you usually do 30 minutes or more walking? (e.g., walking from place to place for exercise, leisure or recreation) *5 or more times a week* *3–4 times a week* *1–2 times a week* *none* 3 How many times a week do you usually do 30 minutes or more of moderate-intensity physical activity that increases your heart rate or makes you breathe harder than normal? (e.g., carrying light loads, bicycling at a regular pace, or doubles tennis) *5 or more times a week* *3–4 times a week* *1–2 times a week* *none* | 140 | 52,87 | 10th to 12th grade (fairly difficult to read) | 35 | 46 |
| Amount of PA | | | | | | |
| Elaborative Exercise Questionnaire | Considering a 7-day period (a week), how many times on average do you do the following kinds of exercise for more than 15 minutes during your free time (write in each circle the appropriate number).  Strenuous Exercise (heart beats rapidly): Running, jogging, hockey, football, soccer, squash, basketball, cross-country skiing, judo, roller skating, vigorous swimming, vigorous long-distance bicycling.  Mark the average number of times that you perform one or more of the above activities during your typical week.  Moderate Exercise (not exhausting): Fast walking, baseball, tennis, easy bicycling, volleyball, badminton, easy swimming, alpine skiing, popular and folk dancing.  Mark the average number of times that you perform one or more of these activities during your typical week.  Mild Exercise (minimal effort): Yoga, archery, fishing from a river bank, bowling, horseshoes, golf, snowmobiling, easy walking.  Mark the average number of times that you perform one or more of these activities during your typical week. | 152 | 34,80 | College (difficult to read) | 38 | 50 |
| Self-Report on Activity 2  (SR-2) | Over the past week (even if it's not a typical week), how much time did you exercise or were you physically active (e.g. strength training, walking, swimming, gardening or other type of training)? *No time.  Less than 30 minutes per week. 30-60 minutes per week. 1-3 hours per week. More than 3 hours per week.* | 55 | 45,16 | College (difficult to read) | 14 | 18 |
| Japan Collaborative Cohort  (JACC) Questionnaire | How much time per week on average do you spend engaging in sports or physical exercise? *1) At least 5 hours. 2) 3-4 hours. 3) 1-2 hours. 4) Little.* How much time per day on average do you spend walking indoors or outside?  *1) More than 1 hour. 2) 30 minutes - 1 hour.  3) about 30 minutes. 4) Little.* How often did you engage in sports or physical exercise over the past year or two?  *1) Seldom.  2) Sometimes.  3) About once a week.  4) At least twice a week.* | 90 | 69,89 | 8th & 9th grade (plain English) | 23 | 30 |
| Nordic Physical Activity Questionnaire – Short Version  (NPAQ-short) | *Close ended:*  Physical activities in your free time.  The following questions concern how physically active you are in your free time and during transport (including your commute to and from work/school/classes).  On a typical week, how much time do you spend in total on moderate and vigorous physical activities where your heartbeat increases and you breathe faster (e.g. brisk walking, cycling as a means of transport or as exercise, heavy gardening, running or recreational sports).  Only include activities that lasted at least 10 minutes at a time.  *Less than ½ an hour (less than 30 minutes).*  *½ an hour – 1 ½ hours (30-90 minutes).*  *1 ½ - 2 ½ hours (90-150 minutes).*  *2 ½ - 5 hours (150-300 minutes).*  *More than 5 hours (150-300 minutes).*  How much of the time that you spend on physical activities in a typical week, which you indicated above, do you spend in total on vigorous physical activities? This includes activities that get your heart racing, make you sweat and leave you so short of breath that speaking becomes difficult (e.g. swimming, running, cycling at high speeds, cardio training, weight-lifting or team sports such as football).  Only include activities that lasted at least 10 minutes at a time.  *Less than ½ an hour (less than 30 minutes).*  *½ an hour – 1 hours (30-60 minutes).*  *1 - 1 ½ hours (60-90 minutes).*  *1 ½ - 2 ½ hours (90-150 minutes).*  *More than 2 ½ hours (more than 150 minutes).* | 237 | 47,74 | College (difficult to read) | 60 | 78 |
|  | *Open ended:*  Physical activities in your free time.  The following questions concern how physically active you are in your free time and during transport (including your commute to and from work/school/classes).  On a typical week, how much time do you spend in total on moderate and vigorous physical activities where your heartbeat increases and you breathe faster (e.g. brisk walking, cycling as a means of transport or as exercise, heavy gardening, running or recreational sports).  Only include activities that lasted at least 10 minutes at a time.  *Hours pr week. Minutes pr week.*  How much of the time that you spend on physical activities in a typical week, which you indicated above, do you spend in total on vigorous physical activities? This includes activities that get your heart racing, make you sweat and leave you so short of breath that speaking becomes difficult (e.g. swimming, running, cycling at high speeds, cardio training, weight-lifting or team sports such as football).  Only include activities that lasted at least 10 minutes at a time.  *Hours pr week. Minutes pr week.* | 175 | 47,74 | College (difficult to read) | 44 | 57 |
| Total Activity Measure 1 (TAM1) | We'd like to know how active you are. 1 In an average week how many times do you do 'Strenuous' activities? Strenuous activity means any activity that take as much effort as – jogging or running, digging in heavy ground, aerobics classes, vigorous swimming, heavy DIY jobs like sawing thick or long pieces of wood, football, circuit training, squash... *'Strenuous activity' times a week.* 1b If you do a ‘strenuous’ activity, how many minutes do you do it for on average? 'Strenuous activity' minutes each time. 2 In an average week how many times do you do ‘moderate’ activities? Moderate activity means any activity that take as much effort as – brisk walking, housework, carrying a light shopping bag on level ground, mowing the lawn, general DIY like painting and decorating, easy swimming, easy cycling, ballroom dancing... *'Moderate activity' times a week.* 2b If you do a moderate activity, how many minutes do you do it for on average? *'Moderate activity' minutes each time.* 3 In an average week how many times do you do any mild activities? Mild activity means any activity that take as much effort as – easy walking, very light housework, browsing in shops, slow dancing, bowls, river fishing, golf, hand weeding in the garden... *'Mild activity' times a week.* 3b If you do a 'mild' activity, how many minutes do you do it for on average? *'Mild activity' minutes each time.* Please check that you have put a number in all 6 boxes, even if it is a 0. Thank you for your help. | 259 | 56,69 | 10th to 12th grade (fairly difficult to read) | 63 | 82 |
|  | TAM 2  We’d like to know how active you are. 1 In an average week how many times do you do 'Strenuous' activities? Strenuous activity means any activity that take as much effort as – jogging or running, digging in heavy ground, aerobics classes, vigorous swimming, heavy DIY jobs like sawing thick or long pieces of wood, football, circuit training, squash... *'Strenuous activity' times a week.* 1b For how many minutes in total do you do strenuous activities? *'Strenuous activity' total minutes.* 2 In an average week how many times do you do ‘moderate’ activities? Moderate activity means any activity that take as much effort as – brisk walking, housework, carrying a light shopping bag on level ground, mowing the lawn, general DIY like painting and decorating, easy swimming, easy cycling, ballroom dancing... *'Moderate activity' times a week.* 2b For how many minutes in total in an average week do you 'mild' activities? 3 In an average week, how many times do you do any mild activities? Mild activity means any activity that take as much effort as – easy walking, very light housework, browsing in shops, slow dancing, bowls, river fishing, golf, hand weeding in the garden... *'Mild activity' times a week.* 3b For how many minutes in total in an average week do you do mild activities? *'Mild activity' total minutes.* Please check that you have put a number in all 6 boxes, even if it is a 0. Thank you for your help. | 244 | 55,31 | 10th to 12th grade (fairly difficult to read) | 59 | 77 |
| Cohort of Norway  (CONOR) Instrument | How has your physical activity during leisure time been over the last year?  Think of your weekly average for the year. Time spent going to or from work counts as leisure time.  Hours per week: None/Less than 1/1-2/3 or more.  Light activity  (not sweating or out of breath).  Hard physical activity  (sweating/out of breath).  Please note physical activity during the past year in your spare time. What does describe you better?  If activity varies between summer and wintertime, note a mean value.  (Tick one only)  Reading, watching TV or any other sedentary activity?  Walking, cycling, or other activity, other for at least 4 hours a week? (Count also walking back and forth from work)  Light sports, heavy gardening? (At least 4 hours per week).  Hard exercise, competitive sports? Regularly and several times a week. | 132 | 67,44 | 8th & 9th grade (Plain English) | 33 | 43 |
| General exercise participation/physical activity involvement questions | | | | | | |
| Self-Report on Activity 1  (SR-1) | I exercise regularly. *1 2 3 4 5* | 8 | N/A | N/A | 2 | 3 |
| Single-item screening question to identify physical inactivity | As a rule, do you do at least half an hour of moderate or vigorous exercise (such as walking or a sport) on five or more days of the week?  *Yes. No.* | 32 | 72,05 | 7th grade (fairly easy to read) | 8 | 10 |
| St. Louis Working Hearts Program single exercise question | Do you currently participate in any regular activity or program (either on your own or in a formal class) designed to improve or maintain your physical fitness? *Yes. No.* | 29 | 32,16 | College (difficult to read) | 6 | 8 |
| PA questions of Brief Health, Fitness and Spirituality Survey | In a typical week, do you do any vigorous-intensity sports, fitness, or recreational activities that cause large increases in breathing or heart rate like running or basketball for at least 10 minutes continuously?  *Yes. No.*  In a typical week, do you do any moderate-intensity sports, fitness, or recreational activities that cause a small increase in breathing or heart rate such as brisk walking, bicycling, swimming, or volleyball for at least 10 minutes continuously?  *Yes. No.*  Do you regularly engage in muscle strengthening activity (such as push-ups, sit-ups, yoga or weight lifting) as a form of exercise?  *Yes. No.* | 92 | 23,98 | College graduate (very difficult to read) | 23 | 31 |
| Self-reported level of PA compared with peers | | | | | | |
| Compared to Peers Question | Would you say that you are physically more active, less active, or about as active as other persons your age? *Elaboration:* Is that a lot more or a little more/a lot less or a little less active? | 36 | 73,42 | 7th grade (fairly easy to read | 9,08 | 11,80 |
| Relative PA Question | Compared to other people your own age, do you think you are... *- Much more active*  *- More active*  *- About as active* *- Less active* *- Much less active* | 25 | 88,91 | 6th grade (easy to read) | 6,30 | 8,20 |
| Categorical descriptions of PA levels | | | | | | |
| The Seven-level Single-Question Scale for Self-Reported Leisure Time Physical Activity  (SR-PA L7) | Which of the following descriptions best corresponds to your physical activity at the moment?  I do not move more than is necessary in my daily routines/chores.  I go for casual walks and engage in light outdoor recreation 1-2 times a week.  I go for casual walks and engage in light outdoor recreation several times a week.  I engage, 1-2 times a week, in brisk physical activity (e.g. yard work, walking, and cycling) to the point of perspiring and some degree of breathlessness.  Several times a week (3-5), I engage in brisk physical activity (e.g. yard work, walking, and cycling) to the point of perspiring and some degree of breathlessness.  I do keep-fit exercises several times a week in a way that causes rather strong shortness of breath and sweating during the activity.  I participate in competitive sports and maintain my fitness through regular training. | 144 | 54,03 | 10th to 12th grade (fairly difficult to read) | 36 | 47 |
| Self-report scale to assess habitual physical activity | If you think about the past 6 months, which of the following descriptions best pictures your physical activity?  Mostly resting, hardly any activity.  Mostly sitting. You are usually doing things in a seated position, reading and watching TV. Your only physical activities relate to activities of daily living (grooming, dressing).  Light physical activity. You are doing light housework (e.g., preparing food, dusting) or light gardening or going for a walk two to three times a week.  Moderate physical activity about 3 hours a week. You are doing common housework (e.g., vacuum cleaning/sweeping floors, lawnmowing) or going for longer walks (at least 2 km) or cycling.  Moderate physical activity at least 4 hours a week or heavier physical activity up to 4 hours a week (daily more than 30 minutes). You are doing moderate physical activities (see before) for at least 4 hours or physically exercise 1–2 hours a week or doing heavy gardening/housework or home maintenance involving some breathlessness and sweating.  You are engaging in active sports several times a week, which makes you heavily sweat and breathless during the exercise or you are doing heavy gardening or leisure time activities (at least 3 hours a week).  You are participating in competitive sports. | 203 | 51,79 | 10th to 12th grade (fairly difficult to read) | 51 | 67 |
| PA categories | Single response from eight descriptors (PA8):  1 I do not exercise/walk regularly now and I do not intend to start in the near future.  2 I do not exercise/walk regularly but I have been thinking of starting.  3 I am trying to start to exercise or walk or I exercise/walk infrequently.  4 I am doing vigorous physical activity less than three times per week or moderate physical activity less than five times per week.  5 I have been doing moderate physical activity that accumulates to at least 30 minutes per day at least 5 days per week for 1-6 months.  6 I have been doing moderate physical activity that accumulates to at least 30 minutes per day at least 5 days per week for 7 or more months.  7 I have been doing vigorous physical activity at least 20 minutes a day 3-5 days per week for 1-6 months.  8 I have been doing vigorous physical activity at least 20 minutes a day 3-5 days per week for 7 or more months. | 166 | 70,09 | 7th grade (fairly easy to read) | 40 | 52 |
|  | Single response from five descriptors (PA5):  1 I don’t exercise or walk regularly now and I don’t plan to start in the near future.  2 I don’t exercise or walk regularly now but I’ve been thinking about starting.  3 I'm doing moderate physical activity fewer than five times a week or vigorous activity fewer than three times a week.  4 I‘ve been doing moderate physical activity 5 or more days a week, or vigorous activity at least 3 days a week, for the last 1 to 6 months.  5 I’ve been doing moderate physical activity 5 or more days a week or vigorous activity at least 3 days a week, for 7 months or longer. | 109 | 68,29 | 8th & 9th grade (plain English) | 27 | 36 |
| Absolute PA Question | What best describes your activity level?  Vigorously active for at least 30 minutes, 3 times per week.  Moderately active for at least 3 times per week.  Seldom active, preferring sedentary activities. | 34 | 57,06 | 10th to 12th grade (fairly difficult to read) | 9 | 11 |
| Job Related Activity | How much hard physical work is required on your job? Would you say a great deal, a moderate amount, a little or none? | 23 | 84,81 | 6th grade (easy to read) | 6 | 8 |
| Main Daily Activity | How much hard physical work is required in your main daily activity? Would you say a great deal, a moderate amount, a little or none? | 25 | 72,32 | 7th grade (fairly easy to read) | 6 | 8 |
| Godin and coworkers simple self-administered instrument  (GCSSI) | How often did you participate in one or more physical activities of 20 to 30 minutes duration per session during your leisure time within the past 6 months?  *1 Not at all.*  *2 Less than once a month.*  *3 About once a month.*  *4 About two or three times a month.*  *5 About one to two times a week.*  *6 Three or more times a week.* | 66 | 54,54 | 10th to 12th grade (fairly difficult to read) | 17 | 22 |
| The Stanford Leisure-Time Activity Categorical Item  (L-CAT) | During the past month, which statement best describes the kinds of physical activity you usually performed during your FREE TIME (i.e., recreational or leisure time)?  1 I did not engage in much physical activity. I mostly did things like watching television, reading, playing cards, or playing computer games. Only occasionally, no more than once or twice a month, did I do anything more active such as getting outdoors for a walk or playing tennis.  2 Once or twice a week, I engaged in light activities such as getting outdoors on the weekends for a walk. Or I did some light chores around the house such as sweeping floors or vacuuming.  3 About three times a week, I did some moderate activity such as brisk walking, swimming, or riding a bike for about 15–20 minutes each time. Or about once a week, I did some moderately difficult chores such as raking, washing windows, or mowing the lawn for about 45–60 minutes. Or about once a week, I played sports such as doubles tennis or basketball for about 45–60 minutes.  4 Almost daily, that is five or more times a week, I did some moderate activity such as brisk walking, swimming, or riding a bike for 30 minutes or more each time. Or about once a week, I did some moderately difficult chores or played team sports for 2 hours or more.  5 About three times a week, I engaged in a regular program of physical fitness involving some kind of heavy or vigorous physical activity such as running or riding hard on a bicycle for 30 minutes or more each time. Or I did chores such as heavy gardening or played active sports such as handball or singles tennis for  60 minutes or more each time.  6 Almost daily, that is, five or more times a week, I engaged in a regular program of physical fitness involving some kind of heavy or vigorous physical activity for 30 minutes or more each time. | 331 | 62,20 | 8th & 9th grade (plain English) | 83 | 109 |
|  | *Version 2.2:* During the past month, which statement best describes the kinds of physical activity you usually did? Do not include the time you spent working at a job. Please read all six statements before selecting one.  1 I did not do much physical activity. I mostly did things like watching television, reading, playing cards, or playing computer games. Only occasionally, no more than once or twice a month, did I do anything more active such as going for a walk or playing tennis.  2 Once or twice a week, I did light activities such as getting outdoors on the weekends for an easy walk or stroll. Or once or twice a week, I did chores around the house such as sweeping floors or vacuuming.  3 About three times a week, I did moderate activities such as brisk walking, swimming, or riding a bike for about 15–20 minutes each time. Or about once a week, I did moderately difficult chores such as raking or mowing the lawn for about 45–60 minutes. Or about once a week, I played sports such as softball, basketball, or soccer for about 45–60 minutes.  4 Almost daily, that is five or more times a week, I did moderate activities such as brisk walking, swimming, or riding a bike for 30 minutes or more each time. Or about once a week, I did moderately difficult chores or played sports for 2 hours or more.  5 About three times a week, I did vigorous activities such as running or riding hard on a bike for 30 minutes or more each time.  6 Almost daily, that is, five or more times a week, I did vigorous activities such as running or riding hard on a bike for 30 minutes or more each time. | 293 | 72,38 | 7th grade (fairly easy to read) | 74 | 96 |
| Usual PA Scale | My job requires very hard physical labor (such as digging or loading heavy objects) at least four hours a day. Or I do vigorous activities (jogging, cycling, swimming, etc.) at least three times per week for 30-10 min. or more. Or I do at least one hour of moderate activity such as brisk walking at least four days a week.  My job requires that I walk, lift, carry or do other moderately hard work for several hours per day (day care worker, stock clerk, or busboy/waitress). Or I spend much of my leisure time doing moderate activities (dancing, gardening, walking or housework).  My job requires that I sit at a desk most of the day.  And much of my leisure time is spent in sedentary activities (watching TV, reading, etc.).  And I seldom work up a sweat and I cannot walk fast without having to stop to catch my breath. | 150 | 69,36 | 8th & 9th grade  (plain English) | 38 | 49 |
| Work Leisure Physical Activity Questionnaire  (WLPAQ) | Describe your physical activity at work (even work at home, sick leave at home and studying, for instance in a university).  1 Very light, e.g., sitting at the computer most of the day or sitting at a desk.  2 Light, e.g., light industrial work, sales or office work that comprises light activities.  3 Moderate, e.g., cleaning, staffing at kitchen or delivering mail on foot or by bicycle.  4 Heavy, e.g., heavy industrial work, construction work or farming.  Describe your physical activity at leisure time. If the activities vary between summer and winter, try to give a mean estimate.  1 Very light: almost no activity at all.  2 Light, e.g., walking, nonstrenuous cycling or gardening approximately once a week.  3 Moderate: regular activity at least once a week, e.g., walking, bicycling, or gardening or walking to work 10–30 minutes per day.  4 Active: regular activities more than once a week, e.g., intense walking or bicycling or sports.  5 Very active: strenuous activities several times a week. | 165 | 56,49 | 10th to 12th grade (fairly difficult to read) | 42 | 54 |
| Speedy Nutrition and Physical Activity Assessment (SNAP) | How Active are You? Examples of activity are Walking  Housework Work in the yard or garden Dancing Jobs that require walking, lifting or other hard work Exercise  Are you active for 30 minutes on 5 days of the week? 1 No and I have no plans to be more active. 2 No, but I have been thinking about being more active. 3 Sometimes I am active for 30 minutes but not all the time. 4 Yes, I am active for 30 minutes on 5 days of the week. | 89 | 88,12 | 6th grade (easy to read) | 22 | 29 |
| Stanford Brief Activity Survey (SBAS) | *Appendix Table 1* A If you have no job or regular work, check box A and go on to Appendix table 2. B I spent most of the day sitting or standing. When I was at work, I did such things as writing, typing, talking on the telephone, assembling small parts, or operating a machine that takes very little exertion or strength. If I drove a car or truck while at work, I did not lift or carry anything for more that a few minutes each day. C I spent most of the day walking or using my hands and arms in work that required moderate exertion. When I was at work, I did such things as delivering mail, patrolling on guard duty, doing mechanical work on automobiles or other large machines, house painting, or operating a machine that requires some moderate-activity work of me. If I drove a truck or lift, my job required me to lift and carry things frequently. D I spent most of the day lifting or carrying heavy objects or moving most of my body in some other way. When I was at work, I did such things as stacking cargo or inventory, handling parts or materials, or doing work like that of a carpenter who builds structures or a gardener who does most of the work without machines. E I spent most of the day doing hard physical labor. When I was at work, I did such things as digging or chopping with heavy tools or carrying heavy loads (bricks, for example) to the place where they were to be used. If I drove a truck or operated equipment, my job also required me to do hard physical work most of the day with only short breaks. *Appendix Table 2* F Most of my leisure time was spent without very much physical activity. I mostly did things like watching television, reading, or playing cards. If I did anything else, it was likely to be light chores around the house or yard or some easy-going game like bowling or catch. Only occasionally, no more than once or twice a month, did I do anything more vigorous, like jogging, playing tennis, or active gardening. G Weekdays, when I got home from work, I did few active things, but most weekends I was able to get outdoors for some light exercise—going for walks, playing a round of golf (without motorized carts), or doing some active chores around the house. H Three times per week, on average, I engaged in some moderate activity, such as brisk walking or slow jogging, swimming, or riding a bike for 15–20 minutes or more, or I spent 45 minutes to an hour or more doing moderately difficult chores, such as raking or washing windows, mowing the lawn or vacuuming, or playing games such a doubles tennis or basketball. I During my leisure time over the past year, I engaged in a regular program of physical fitness involving some kind of heavy physical activity at least three times per week. Examples of heavy physical activity are jogging, running, or riding fast on a bicycle for 30 minutes or more; heavy gardening or other chores for an hour or more; active games or sports such as handball or tennis for an hour or more; or a regular program involving calisthenics and jogging or the equivalent for 30 minute or more. J Over the past year, I engaged in a regular program of physical fitness along the lines described in the last paragraph (I), but I did it almost daily—five or more times per week. | 600 | 60,80 | 8th & 9th grade (plain English) | 151 | 197 |
| The six-point scale | 1 Moving only for necessary chores.  2 Walking or other outdoor activities one or two times per week.  3 Walking or other outdoor activities several times per week.  4 Exercising one or two times per week to the point of perspiring and heavy breathing.  5 Exercising several times per week to the point of perspiring and heavy breathing.  6 Keep-fit heavy exercise or competitive sport several times per week. | 69 | 64 | 8th & 9th grade (plain English) | 17 | 23 |
| Occupational Physical Activity Question | When you are at work, which of the following best describes what you do? *Mostly sitting or standing. Mostly walking. Mostly heavy labor or physically demanding work. Do not work. Don’t know/not sure. Refused.* | 35 | 89,90 | 6th grade (easy to read) | 9 | 11 |
| Eurobarometer Survey Question | How often do you exercise, play sport, or engage in other physical activity, such as cycling from one place to another, dancing, gardening, etc.? *Never. Occasionally. Few times a week. Almost daily.* | 32 | 41,48 | College (difficult to read) | 8 | 10 |
| Gothenburg Instrument | Please note physical activity during the past year in your spare time. What does describe you better.  If activity varies between summer and wintertime, note a mean value.  (Tick one only)   1. Reading, watching TV or any other sedentary activity? 2. Walking, cycling, or other activity, other for at least 4 hours a week? (Count also walking back and forth from work.) 3. Light sports, heavy gardening? (At least 4 hours per week.) 4. Hard exercise, competitive sports? Regularly and several times a week | 80 | 63,36 | 8th & 9th grade (plain English) | 20 | 26 |

* = Only the main text of the questionnaires was used to calculate the level of readability. The text written in italics was excluded from the analysis.

PA = Physical Activity
